# A study on hepatopathic, dyslipidemic and immunogenic properties of fructosylated-HSA-AGE and binding of autoantibodies in sera of obese and overweight patients with fructosylated-HSA-AGE

**DOI:** 10.1371/journal.pone.0216736

**Published:** 2019-05-22

**Authors:** Asif Zaman, Zarina Arif, Moinuddin, Kafil Akhtar, Wasif Mohammad Ali, Khursheed Alam

**Affiliations:** 1 Department of Biochemistry, J.N. Medical College, Faculty of Medicine, Aligarh Muslim University, Aligarh, Uttar Pradesh, India; 2 Department of Pathology, J.N. Medical College, Faculty of Medicine, Aligarh Muslim University, Aligarh, Uttar Pradesh, India; 3 Department of Surgery, J.N. Medical College, Faculty of Medicine, Aligarh Muslim University, Aligarh, Uttar Pradesh, India; University of Pennsylvania, UNITED STATES

## Abstract

Over consumption of fructose may lead to obesity and dyslipidemia and cause fructosylation-induced alterations in the structure and function of proteins. The aim of this study was to investigate the role of fructosylated-HSA-AGE in the pathogenesis of fatty liver (NAFLD and NASH) by biochemical, immunological and histological studies. Immunogenicity of fructosylated-HSA-AGE was probed by inducing antibodies in rabbits. Fructosylated-HSA-AGE was found to be highly immunogenic. Furthermore, fructosylated-HSA-AGE caused mild fibrosis with steatosis and portal inflammation of hepatocytes in experimental animals. Liver function test and dyslipidemic parameters in immunized animals were also found to be raised. Ultrasonography, which should form part of the assessment of chronically raised transaminases, shows fatty infiltration. Interestingly, alanine aminotransferase (ALT), aspartate aminotransferase (AST), bilirubin, total cholesterol (TC) and triglyceride (TG) profiles confirms USG images of overweight, obese patients. Thus, present study demonstrates that fructosylated-HSA-AGE is hepatotoxic, immunologically active and may cause dyslipidemia.

## Introduction

Fructose is a reducing and exceptionally lipogenic monosaccharide and invigorates triglyceride synthesis. Its utilization has been connected to obesity, insulin resistance, dyslipidemia, weakened glucose resistance and hypertension [1]. The hepatic metabolism of fructose is different from glucose in that it is insulin independent. In addition, the passage of fructose into glycolysis through fructose-1-phosphate bypasses the primary glycolysis control step catalyzed by phosphofructokinase [2,3,4]. Overweight and obesity has been attributed to unusual or unnecessary fat deposition in the body tissues that debase wellbeing and may prompt either NAFLD or NASH [3]. Body fat distribution in specific regions with abdominal fat may result in elevation of liver enzymes [5,6].NAFLD is the most well-known clarification for liver aminotransferase elevation in obesity [7,8]. The liver is made out of parenchymal cells (hepatocytes) and non-parenchymal cells (liver sinusoidal endothelial cells, Kupffer cells and hepatic stellate cells) [9]. Accumulation of fat in the cytoplasm of the hepatocytes characterized by a micro- and macro-vesicular steatosis, fibrosis and inflammation are the common indications of NAFLD beginning, which may prompt NASH [10]. Past examinations in rodents have shown different histological changes in liver tissue after fructose utilization; these incorporate inflammation in the periportal locales and macrovesicularsteatosis in the periportal zone [2]. In addition, high fructose utilization may add to NAFLD pathologic process since fructose-incited ATP consumption advances to hepatic necro-inflammation [1]. Fructose can cause oxidative stress to the liver by draining hepatic energy supplies. It has been shown that normal human subjects and NASH patients exhibita similar exhaustion in hepatic ATP levels after infusion of fructose, however recuperation of ATP levels after fructose consumption was slower in patients with NASH contrasted with healthy human subjects [4].A couple of direct (formation of advanced glycation end products) and backhanded (induction of the metabolic syndrome) components may contribute to fructose-induced NAFLD [11]. Furthermore, hyperglycemia and obesity may likewise intensify NAFLD [12].Advancedglycation end products (AGEs) are formed as a result of non-enzymatic binding of reducing sugars with proteins, lipids, and nucleic acids. These macromolecular-AGEs (whether fructated or glucated) are impaired in their structure, function and more susceptible to oxidative damage [13,14,15]. A potential component by which fructose may cause liver damage additionally exists: liver does not use all fructose and a few moieties tie to macromolecules and shape AGEs. Fructose creates multiple times a bigger number of ROS than glucose, which, if not quenched by an antioxidant (like glutathione in liver), can advance hepatocellular damage [16].

Human serum albumin (HSA) is an extracellular heart-shaped three-domain protein that is mainly synthesized by hepatocytes and is the most abundant protein in plasma [17]. Passage of fructose into hepatocytes prompts the fructosylation of cytoplasmic proteins, causing modification and dysregulation of the structure and capacity of these proteins [18]. The accumulation of AGEs have been linked to diabetes [19,20],cirrhosis [21], atherosclerosis [22] and neurodegenerative diseases [23].Fructose-derived AGEs not just advances to the arrangement of cross-linkages between key atoms but also interact with specific receptors on the cell surfaces resulting in unusual intracellular signaling [18]. Receptors for advanced glycationend product (RAGE) are generally present on both parenchymal and non-parenchymal liver cells [9]. The AGE and RAGE interaction have been previously reported to activate intracellular signaling, produce pro-inflammatory cytokines and induce gene expression[24]. Contingent upon the cell and conditions, the AGE-RAGE communication in hepatocytes and hepatic stellate cells can cause expanded generation of ROS and hepatic inflammation. This may enhances cell proliferation and activation, thus playing a role in the progression of hepatic fibrosis [25]. Proof from experimental models and human examinations propose that oxidative stress is the principle factor in the development of NAFLD and NASH progression [25,26]. Ongoing reports recommends aggregation of the N^ε^-carboxymethyllysine (CML) in the liver, which is related with hepatic steatosis and hepatic aggravation in liver of obese people [27,28]. As of late, another strategy has been produced for immunological discovery of fructosylated-AGEs in diabetes and its complications [29]. Further immunohistochemical examinations of fructosylated-AGEs in liver biopsy are required to uncover the job of these injurious AGEs in the progression of liverdiseases. Various examiners have detailed the structural characterization glycated/fructosylated HSA [30,20].

Our laboratory has also reported formation of fructosylated-HSA-AGEs [31].In this study, we have investigated immunogenicity, hepatotoxicity and dyslipidemic properties of native and fructosylated-HSA-AGE in rabbits. Furthermore, circulating autoantibodies against fructosylated-HSA-AGE have been evaluated in sera of obese and overweight patients.

## Materials and methods

### Ethics statement

All experiments on animals were performed according to the instructions of the Council of International Organizations of Medical Sciences for the care and use of laboratory animals. The animal immunization protocols, experimental designs and studies were specifically approved by the Institutional Animal Ethics Committee (IAEC), J.N. Medical College, Aligarh Muslim University, Aligarh vide letter no. 401/GO/Re/S/2001/CPCSEA. The IAEC clearance was approved by the institutional Ethics Committee (IEC) vide letter no. 363/FM. The USG images of liver of healthy human subjects and overweight and obese patients were recorded according to the protocol described in the Helsinki declaration. Five milliliters of fresh blood samples were obtained from healthy volunteers after obtaining their verbal consent. Blood samples from patients were withdrawn after their verbal consent in presence of radiologist.

### Chemicals

Human serum albumin (HSA), sodium dodecyl sulphate (SDS), sodium azide (NaN_3_), dialysis tubing, anti-rabbit and anti-human IgG-alkaline phosphatase conjugates, p-nitrophenyl phosphate (pNPP), Protein A-agarose column, Tween-20 and Freund’s complete & incomplete adjuvants were purchased from Sigma Aldrich, USA. D-Fructose and silver nitrate were obtained from Qualigens, India. Polystyrene flat bottom microtiterELISA plates were from Nunc (Roskilde, Denmark). All other chemicals used in the study used were of highest analytical grade.

### Patients’ selection and sampling

The BMI of obese and overweight patients suspected of fatty liver was recorded and their blood samples were processed for ALT, AST, bilirubin, TC and TG levels. The BMI is an index of weight-for-height and is used to categorize overweight and obesity in adults. It is defined as person's weight in kilograms divided by the square of his height in meters (kg/m^2^). If BMI is 18.5 to <25, the individual is healthy, if BMI is 25.0 to <30, the individual is overweight and if the BMI is 30.0 or more, the individual is obese. The data on subjects included in this study have been summarized in Table 1. The complement proteins were inactivated by heating the sera at 56°C for 30 min. All samples were stored with the addition of 0.1% sodium azide as a preservative in aliquots at -20°C.

**Table 1:** Biochemical features, age and BMI of healthy subjects, overweight and obese patients included in this study.

Parameter	Healthy subjects (n = 10)	Overweight patients (n = 10)	Obese patients (n = 10)
Age (20–35 yr)	28.7 ± 4.37	28.6 ± 4.19	29.3 ± 3.56
BMI (Kg/m^2^)	23.85 ± 0.711	27.91 ± 1.59*	38.51 ± 2.47**
ALT (IU/L)	9.7± 1.706	16.75± 4.46*	17.58± 5.96**
AST (IU/L)	10 ± 1.87	15.25 ± 4.56*	15.83 ± 5.28**
AST/ALT	1.068 ± 0. 304	0.910 ± 0.096	0.877 ± 0.124
TC (mg/dl)	127.3 ±13.47	150.4 ±15.35*	174.8 ±23.33**
TG (mg/dl)	107.2 ± 15.61	152.1±23.25*	163.6 ±28.75**

*p<0.05 compared to healthy individuals.

**p<0.05 compared to healthy individuals.

Data are expressed as mean ± standard deviation (±SD).

BMI (body mass index), ALT (alanine aminotransferase), AST (aspartate aminotransferase), TC (total cholesterol), TG (triglycerides).

### Preparation of fructosylated-HSA-AGE

In our previous study [31], have demonstrated alterationsin the structure of HSA upon fructosylation. Very briefly, HSA (0.015 mM) was incubated with three concentrations of fructose (5, 10 and 20 mM) at 37°C for 10 days in 10 mM phosphate buffered saline (pH 7.4) containing 0.02% sodium azide. At the end of incubation, the unbound fructose was removed by extensive dialysis. HSA (0.015 mM) devoid of fructose and incubation under identical conditions served as control.

### Animal care

New Zealand White (NZW) female rabbits weighing 1–1.5 kg(8–12 weeks) were placed in cages with square mesh with 12 h light and dark cycle. The room temperature was maintained at 28±2 °C and 50% humidity. All animals were given standard diet and water ad libitum.

### Immunization schedule

Rabbits were immunized with native HSA and fructosylated-HSA-AGE as described previously [32]. Briefly, experimental rabbits (n = 4; two for each native HSA and fructosylated-HSA-AGE) were immunized intramuscularly at multiple sites with 250 μg of the antigenin sterile PBS (total volume 1ml) emulsified withequal volume ofFreund's complete adjuvant. After the first dose of antigen, the animals were given weekly injections with same amount of antigen but with Freund’s incomplete adjuvant. Test bleeds were performed every week and checked for ALT, AST, bilirubin, TC and TG levels. Serum was separated from the blood and complement proteins were inactivated by heating the sera at 56°C for 30 min. All samples were stored with the addition of 0.1% sodium azide as a preservative in aliquots at -20°C. After the end of the study the animals were sacrificed, livers were excised stored at -80°C. For histological evaluations, tissues were fixed in 4% paraformaldehyde and paraffin embedded.

### Measurement of ALT, AST, bilirubin, TC and TG in serum/plasma of humans and animals

ALT, AST, bilirubin were measured in sera by standard methods. Total cholesterol (TC) and triglycerides (TG) were estimated by BeneSphera^TM^ kits using LablifeChemMaster RT–1904C analyzer.

### Enzyme linked immunosorbent assay (ELISA)

Direct binding ELISA was performed [33] to determine the titre of experimentally induced antibodies against native HSA and fructosylated-HSA-AGE,and also for autoantibodies against fructosylated-HSA-AGE in obese and overweight patients. Briefly, wells of polysorpimmunoplates were filled with 100 μl of native HSA or fructosylated-HSA-AGE (10 μg/ml) in antigen coating buffer. The plates were placed at 37°C for 2 h followed by overnight incubation at 4°C. Each sample was assayed in duplicate and half of the plate, devoid of antigen, served as control. Next, the plates were washed with TBS-T (20 mMTris, 2.68 mMKCl, 150 mMNaCl, pH 7.4, containing 0.05% Tween-20) to remove the unbound antigen. Unoccupied sites were blocked with 2% fat free milk in TBS (10 mMTris, 150 mMNaCl, pH 7.4) for 4–6 h at 37°C. After incubation, the plates were washed 5–6 times with TBS-T. Test serum, serially diluted in TBS- T, was added to each well (100 μl/well) and incubated for 2 h at 37°C and then overnight at 4°C. The plates were washed three times with TBS-T and twice with distilled water. Bound antibodies were assayed with anti-rabbit/anti-human IgG-alkaline phosphatase conjugate using p-nitrophenyl phosphate as substrate. The developed color was read at 405 nm on a microplate reader. Results were expressed as a mean of difference of absorbance values in test and control wells (A_test_− A_control_).

### Purification of IgG

Immunoglobulin G (IgG) was isolated from preimmune and immune rabbits’seraas well from the sera of obese and overweight patients on protein A-Agarose column [32]. The homogeneity of isolated IgG was checked on 10% sodium dodecyl sulfate-polyacrylamide.

### Inhibition ELISA

Antigen-binding specificity of the antibodies was determined by inhibition ELISA[34]. The plates were coated with 100μl of antigen (native HSA or fructosylated-HSA-AGE, 10 μg/ml) for 2 h at 37°C and overnight at 4°C. Varying amount of inhibitors (0–20 μg/ml) were mixed with a constant amount of antiserum orpurified IgG. The mixture was incubated at room temperature for 2 h and overnight at 4°C. The immune complex thus formed was added to the wells, in place of serum. The remaining steps were same as in direct binding ELISA. Percent inhibition was calculated using the following formula:

$$\%\;inhibition\;=\;1-\left(\frac{A_{inhibited}}{A_{uninhibited}}\right)\times 100$$

### Band shift assay

Antigen-antibody interaction was confirmed by visual detection of immune complex formation [35]. A constant amount of native HSA or fructosylated-HSA-AGE was mixed with varying amounts of purified anti-fructosylated-HSA-AGE IgG in PBS for 2 h at 37°C and overnight at 4°C. At the end of incubation the antigen-antibody complex was then electrophoresed on 10% SDS–polyacrylamide gel for 4 h at 80 V and the protein bands were visualized by silver staining.

### Histopathology of liver sections from immunized rabbits

The anatomical changes in the liver sections of immunized rabbits were studied after staining with hematoxylin and eosin [36]. Slices of liver sections were fixed in 10% formalin. Paraffin blocks of the liver were fixed to the holder and 4 μm thick sections were cut. The sections were then removed with xylene and hydrated with decreasing alcohol levels. The sections were finally dehydrated with rising alcohol levels and cleaned with xylene before being mounted in a mixture of distyrene, plasticizer and xylene (DPX) for optical microscope observation.Photomicrographs were taken with a digital camera mounted on Olympus microscope.

### Scanning Electron Microscopy (SEM) of rabbit liver sections

Sample blocks for SEM were processed as described [37]. Blocks were fixed in 3% glutaraldehyde buffer (pH 7.2), with the help of a tissue processor, dehydrated with ethanol and solid dried in CO_2_. Each tissue block was divided into two equal halves and each half was attached to the newly exposed surface facing upwards. Spotter coater was used to gold coat the stubs. A scanning electron microscope (JEOL JSM-6510LV) with an acceleration voltage of 5 kV was used for capturing SEM images.

### Statistical analysis

Data are expressed as mean ± standard deviation (±SD). Statistical significance of the results was evaluated by Student’s t-test using GraphPad Prism7 Software. A p-value of <0.05 was considered as significant.

## Results

### Characterization of fructosylated-HSA-AGE

Our previous study [31] on fructosylated-HSA-AGE has shown hyperchromicity, quenching of tryptophan fluorescence, changes in secondary and tertiary structures, increase in molecular mass as well as melting temperature, generation of fluorescent and non-fluorescent AGEs and formation of amyloidogenic aggregates.

### Immunogenicity of native HSA and fructosylated-HSA-AGE

**Fig 1:**
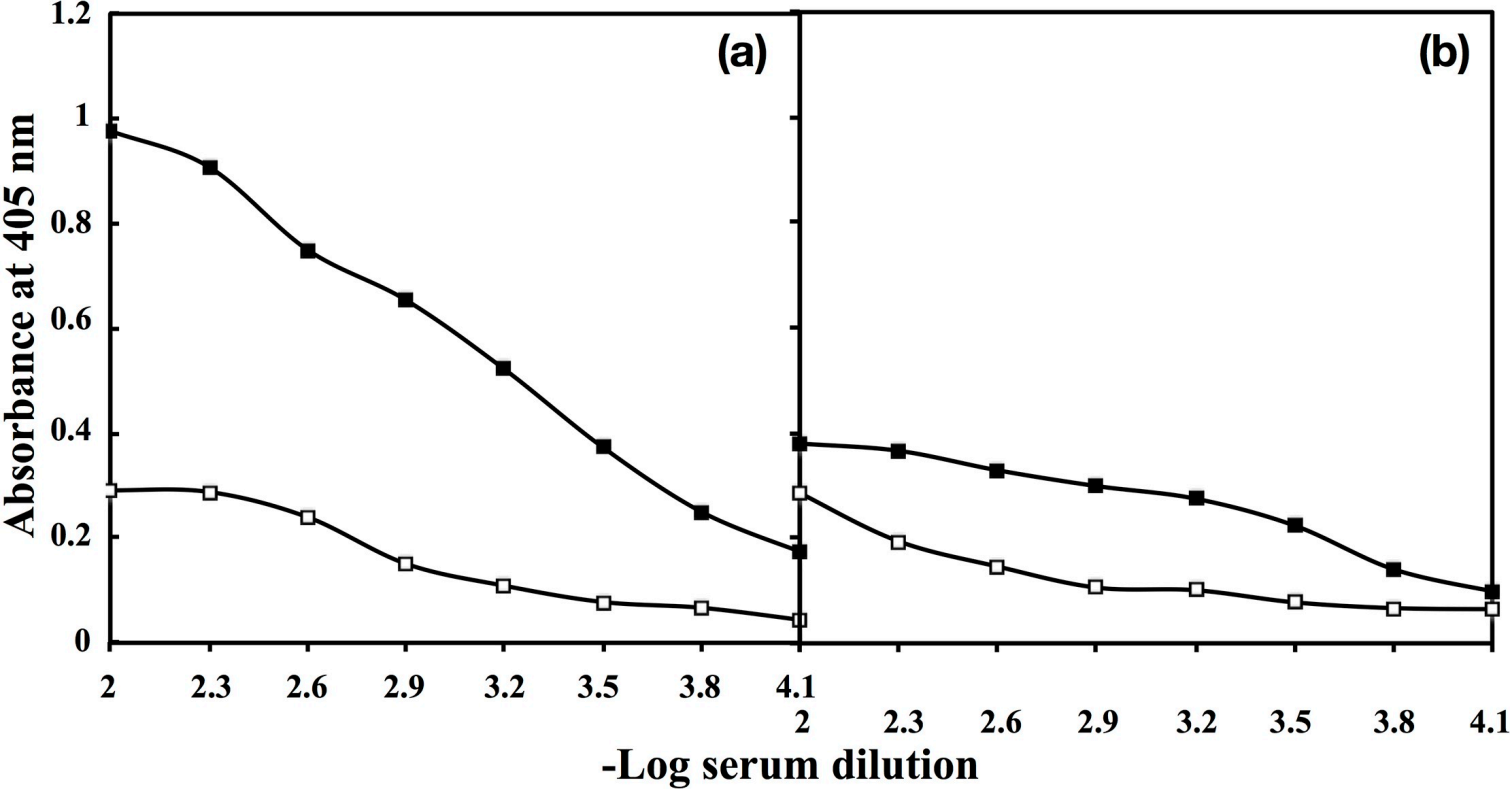
Direct binding ELISA of fructosylated-HSA-AGE (a) and native HSA (b) with pre-immune (□) and immune (■) sera.Microtitre plates were coated with the respective antigens (10 μg/ml).

Native HSA and fructosylated-HSA-AGE were injected into rabbits andtitre of the induced antibodies was determined by direct binding ELISA. Anti-fructosylated-HSA-AGE antibodies showed antibody titre>1:6400 (Fig 1A) as compared to <1:1600 titre shown by anti-native HSA antibodies (Fig 1B). Preimmune serum showed quite low binding with either immunogens. The results suggest that fructosylated-HSA-AGE is a potent immunogen.

### Specificity of induced antibodies against native HSA and fructosylated-HSA-AGE

**Fig 2:**
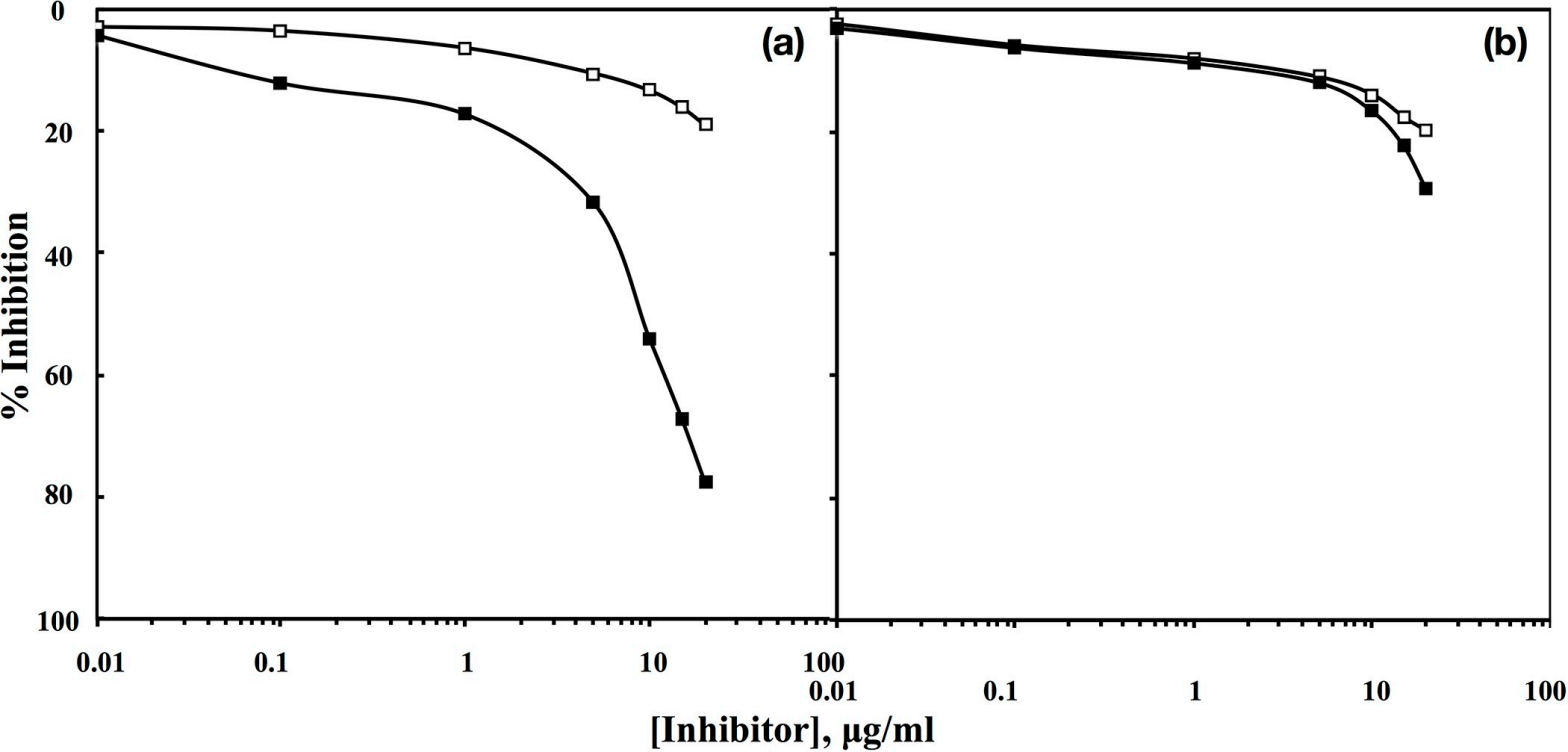
Inhibition ELISA of fructosylated-HSA-AGE (a) and native HSA (b) with pre-immune (□) and immune (■) sera. Native HSA and fructosylated-HSA-AGE (10 μg/ml) were used as a coating antigen as well as inhibitors, respectively.

Specificity of the induced antibodies against respective immunogens was determined by inhibition ELISA. A maximum of 77.6% inhibition in anti-fructosylated-HSA-AGE antibodies binding was observed when fructosylated-HSA-AGE was used as inhibitor (Fig 2A); 50% inhibition was seen at 9 μg/ml of fructosylated-HSA-AGE. Under identical conditions anti-native HSA showed only 29.4% inhibition in binding when native HSA was used as an inhibitor (Fig 2B).

### Isolation of IgG from rabbits’ sera

**Fig 3:**
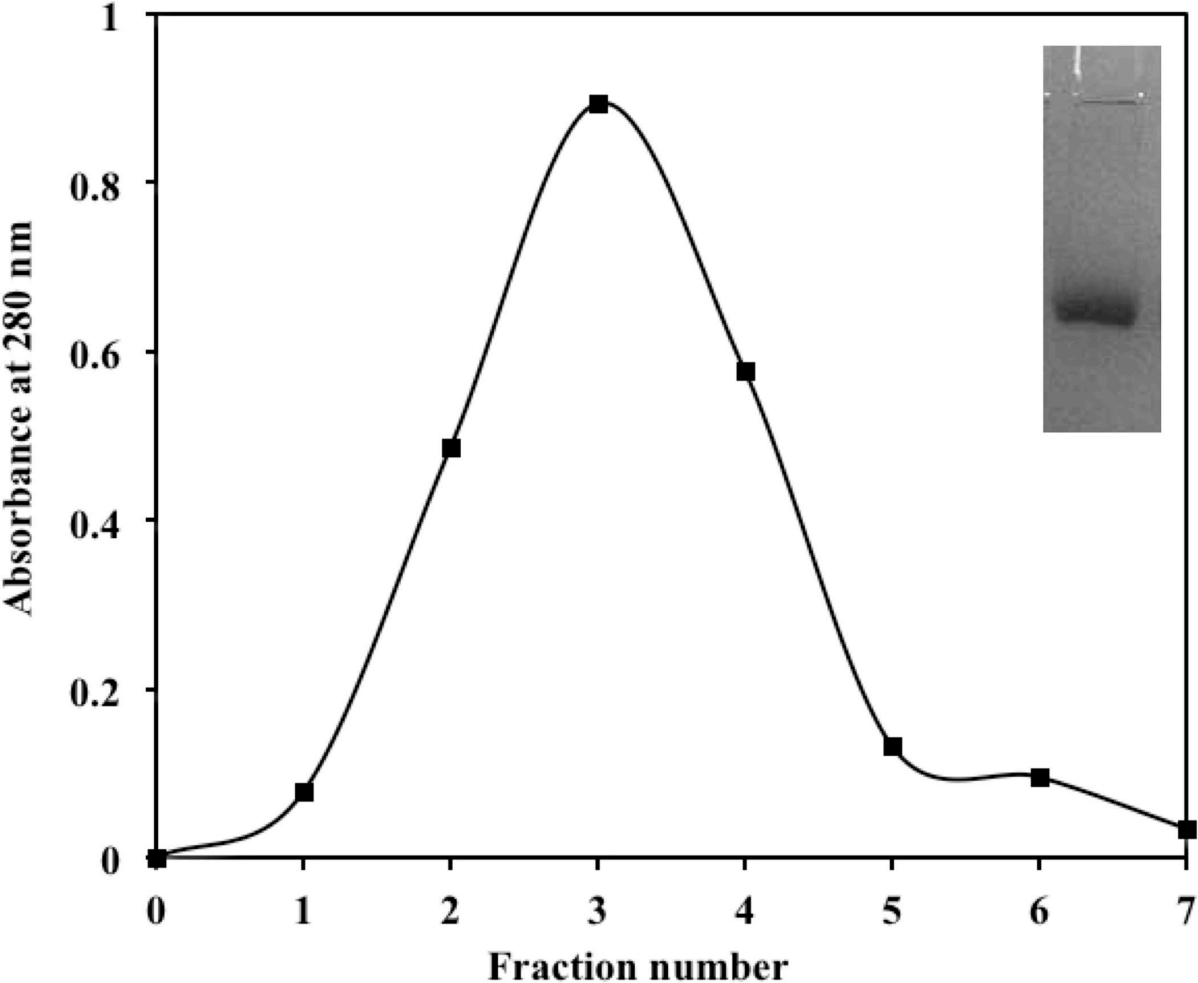
Elution profile of anti- fructosylated-HSA-AGE-IgG on protein-A agarose affinity column. Inset: SDS-PAGE photograph of purified IgG on 10% polyacrylamide gel.

IgG was isolated from pre-immune and immune rabbit sera by affinity chromatography on protein A-agarose column. Fig 3 shows the elution profile of IgG fromfructosylated-HSA-AGE antiserum. The homogeneity of isolated IgG has been shown in inset to Fig 3.

### Specificity of IgG isolated from anti-sera of native HSA and fructosylated-HSA-AGE

**Fig 4:**
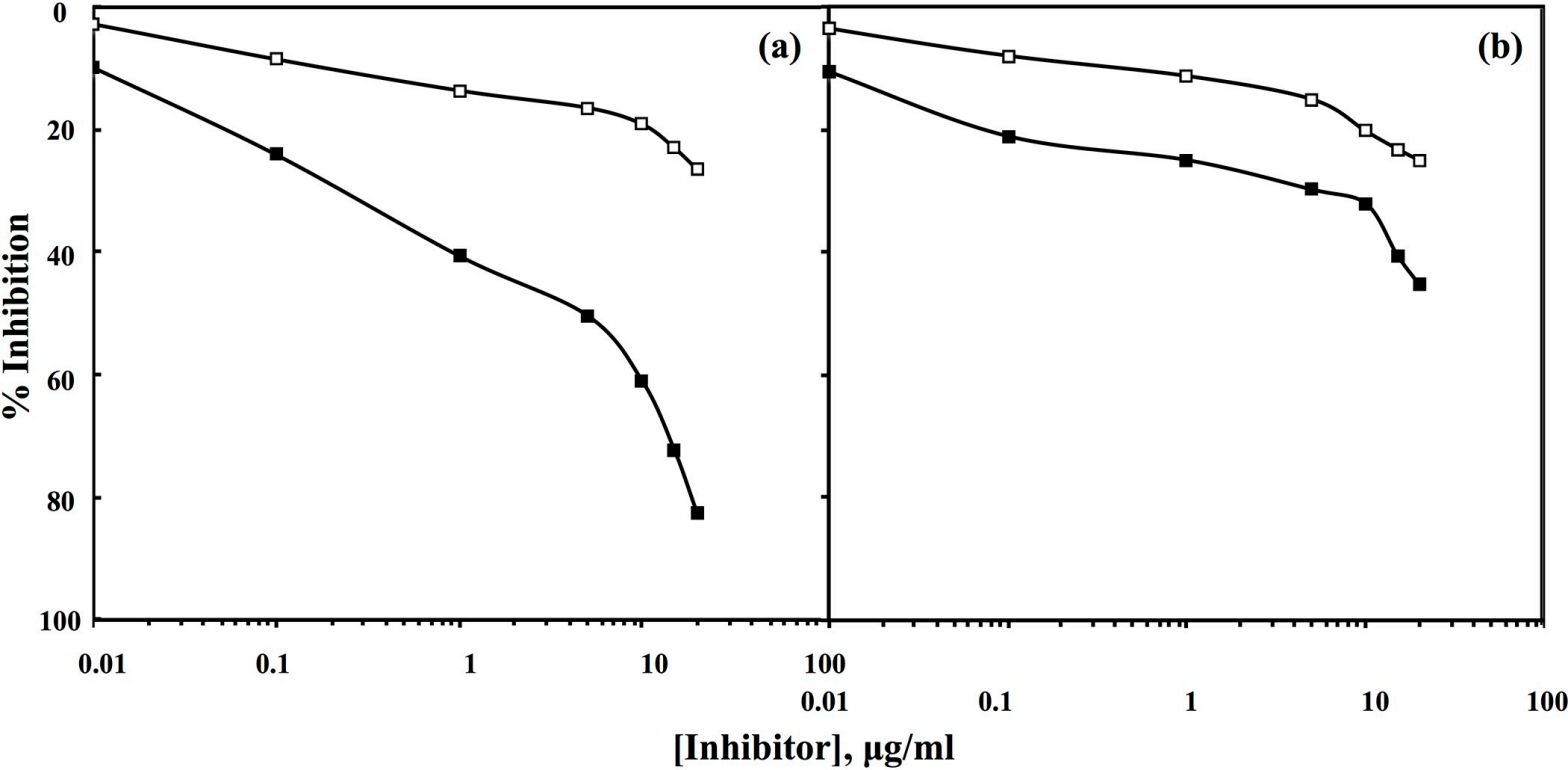
Inhibition ELISA of fructosylated-HSA-AGE (a) and native HSA (b) with respective pre-immune (□) and immune (■) IgGs. Native HSA and fructosylated-HSA-AGE (10 μg/ml) were used as a coating antigen as well as inhibitors, respectively.

Specificity of IgG purified from antiserum of fructosylated-HSA-AGE was evaluated by inhibition ELISA. A maximum of 82.7% inhibition in anti- fructosylated-HSA-AGE IgG binding was observed when fructosylated-HSA-AGE was used as inhibitor (Fig 4A);50%inhibition was seen at 5 μg/ml of fructosylated-HSA-AGE. Under identical conditions anti-native HSA IgG showed only 45.4% inhibition in binding when native HSA was used as an inhibitor (Fig 4B).

### Cross reactivity of anti-fructosylated-HSA-AGE IgG with other native and modified protein inhibitors

The data shown in Table 2 suggests that though anti-fructosylated-HSA-AGE IgG antibodies have highest binding against the immunogen but epitopes on fructosylated-IgG, histone and BSA have some similarity with typical epitopes of fructosylated-HSA-AGE.

**Table 2:** Cross-reactivity of anti-fructosylated-HSA-AGEIgG with other inhibitors.

Inhibitor	Maximum percent inhibition at 20 μg/mL	Concentration for 50% inhibition (μg/mL)
Fructosylated-HSA	82.7	5
Native HSA	57.3	17.3
Fructosylated-human IgG	54.5	17
Native human IgG	30.9	-
Fructosylated-histone	43.7	-
Native histone	30.6	-
Fructosylated-BSA	45.9	-
Native BSA	25.8	-

### Gel retardation assay

**Fig 5:**
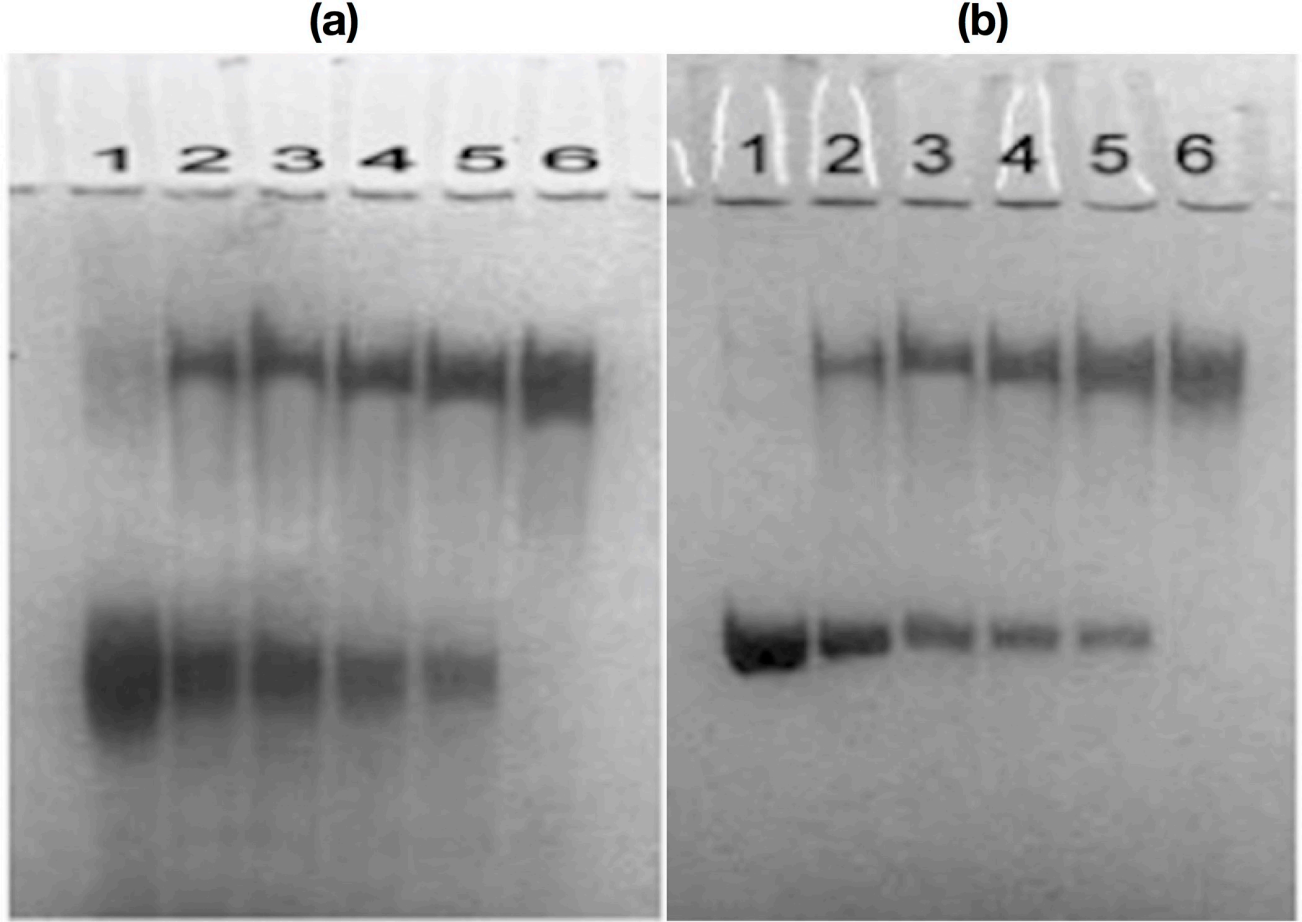
Band shift assay of anti- fructosylated-HSA-AGE IgG binding to fructosylated-HSA (a) native HSA (b) Electrophoresis was performed on 10% SDS–PAGE for 4 hrs at 80 V. **(a)**Fructosylated-HSA-AGE (25 μg/ml, lane 1) was incubated with 10, 20, 30 and 40 μg anti- fructosylated-HSA-AGE IgG, respectively (lanes 2–5) for 2 h at 37 °C and overnight at 4 °C under identical conditions. Lane 6 contains 40 μg anti- fructosylated-HSA-AGE IgG.**(b)** Native HSA (25 μg/ml, lane 1) was incubated with 10, 20, 30 and 40 μg anti- fructosylated-HSA-AGE IgG, respectively (lanes 2–5) for 2 h at 37 °C and overnight at 4 °C. Lane 6 contains 40 μg anti- fructosylated-HSA-AGE IgG.

For the visual detection of the immunogen-antibody interaction, gel retardation assay was performed in polyacrylamide gel containing SDS. The pattern of bands in Fig 5A clearly suggests strong interaction between fructosylated-HSA-AGE (antigen) and anti- fructosylated-HSA-AGE IgG (antibody). There was corresponding increase in mass of immune complex accompanied by retardation in mobility and a proportional decrease in the intensity of unbound antigen (Fig 5A, lane 2–5). However, under identical experimental conditions native HSA incubated with the same amount of anti- fructosylated-HSA-AGE IgG also showed some high molecular weight immune complex and slight decrease in the intensity of the unbound antigen but significantly less as compared to fructosylated-HSA-AGE incubated with the same amount of anti- fructosylated-HSA-AGE IgG (Fig 5B). This indicates that though the induced antibodies against fructosylated-HSA-AGE are specific but they are also recognizing the old epitopes on native HSA.

### Effect of fructosylated-HSA-AGE administration on markers of liver function and dyslipidemia

Weekly injections of 250 μg of fructosylated-HSA-AGE for 7 weeks caused slight changes in ALT, AST, bilirubin, TC and TG level (Table 3). We decided to continue with the weekly dose for 7 weeks more. At the end of 14 weeks the parameters of liver function (ALT, AST, bilirubin) and dyslipidemia (TC and TG) showed further increase (Table 3).

**Table 3:** Effect of fructosylated-HSA-AGE administration on ALT, AST, bilirubin, TC and TG.

Parameter (Reference range)	7 weeks		14 weeks	
	Native HSA	Fructosylated-HSA-AGE	Native HSA	Fructosylated-HSA-AGE
ALT (2–15 I.U./L)	9±1.41	17.25±4.59*	11.25±1.06	40±4.24^#^
AST (2–20 I.U./L)	6.75±0.353	21.75±3.18*	7.95±0.919	39.75±1.76^#^
Bilirubin (0.1–1.2mg/100ml)	0.15±0.07	0.2	0.4±0.141	0.65±0.070
Total cholesterol (<150 mg/dl)	89.5±2.12	117.5±4.94*	115±15.55	231±11.31^#^
Triglycerides (<150 mg/dl)	66±11.3	135±12.72*	87±12.72	185±5.65^#^

*p< 0.05 versus native HSA after 7 weeks.

#p< 0.05 versus native HSA after 14 weeks.

Data are expressed as mean ± standard deviation (±SD).

ALT (alanine aminotransferase), AST (aspartate aminotransferase), TC (total cholesterol), TG (triglycerides).

### Histopathology of liver sections from immunized rabbits

**Fig 6:**
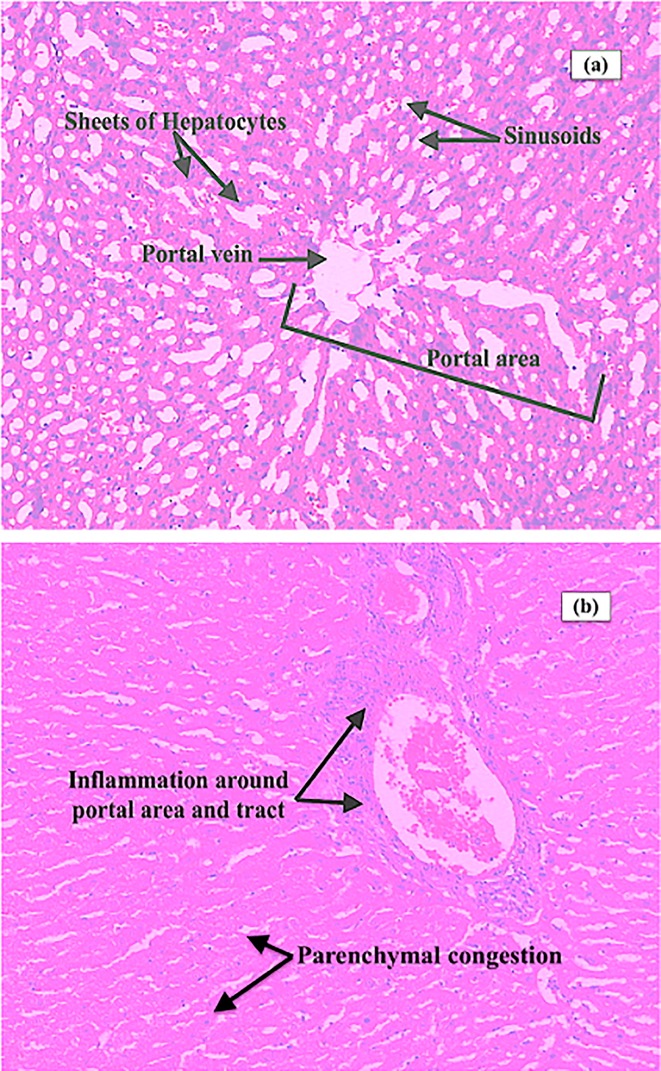
Photographs of the rabbit liver sections stained with hematoxylin and eosin. (a) native HSA at 100X magnification; (b) fructosylated-HSA-AGE at 100X magnification.

Liver section of rabbit with native HSA showed normal morphology (Fig 6A). However, section taken from rabbit liver injected with fructosylated-HSA-AGE showed congested portal vein with periportal inflammation, sinusoidal and parenchymal congestion and mild fibrosis along the portal tract (Fig 6B). Furthermore, band of lymphocytic infilterate in the hepatic parenchyma, binucleation of hepatocytes with micro and macro-vesicular steatosis was also observed (Fig 6B).

### Scanning Electron Microscopy of liver sections

**Fig 7:**
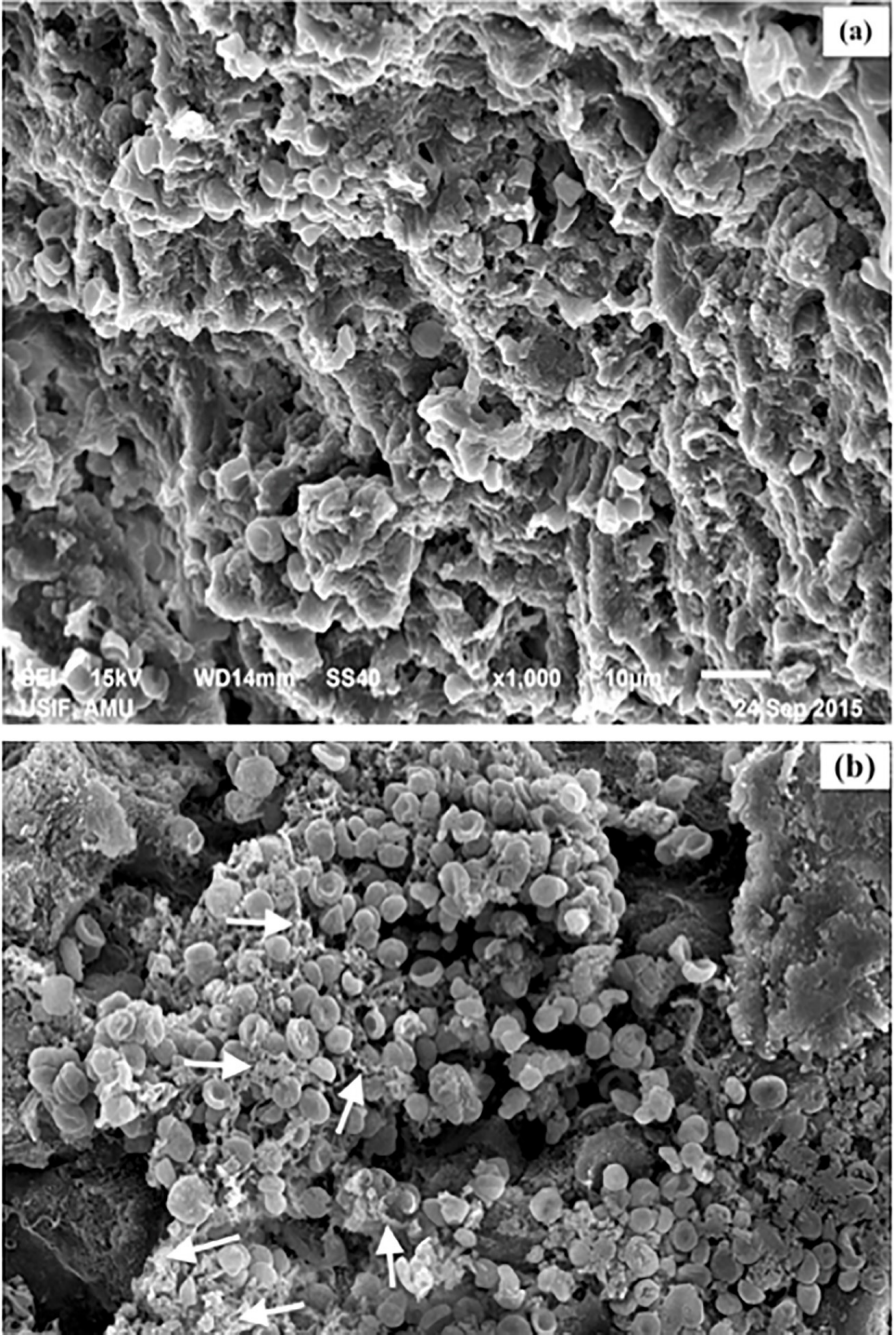
SEM images of the rabbit liver sections. (a) No collagen fibers was seen in the extracellular matrix (ECM) of liver section obtained from native HSA immunized animal: 1000X magnification (b) thick collagen fibers were present in the ECM around hepatocytes (white arrows): of liver section obtained from fructosylated-HSA-AGE immunized animal: 1000X magnification.

It is widely held that persistent hepatic inflammation can trigger the activation of stellate cells and production of collagen in excess, which may deposit around the portal and central vein area. Liver section of rabbit immunized with fructosylated-HSA-AGE showed collagen fibers around hepatocytes in (Fig 7B) as compared to liver of rabbit immunized with native HSA (Fig 7A).Furthermore, profused collagen deposits were filled in the extracellular spaces of the liver parenchyma of fructosylated-HSA-AGE treated rabbit.

### Detection of autoantibodies against fructosylated-HSA-AGE in sera of obese and overweight patients

**Fig 8:**
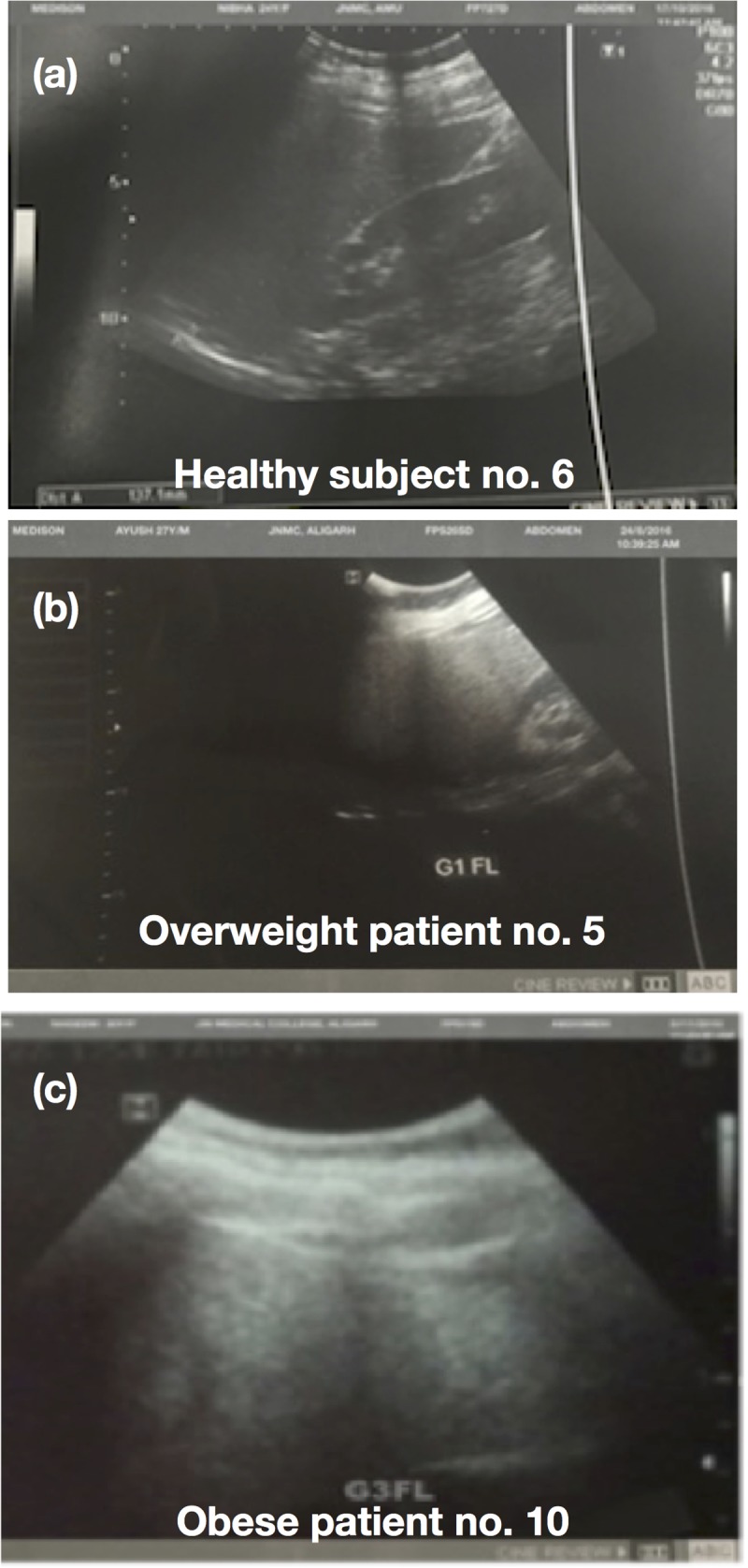
USG images of human liver. (a) healthy subject, (b) overweight patient and (c) obese patient.

The study comprised 10 sera each of healthy subjects, obese patients and overweight patients. Their AGE, BMI, ALT, AST, AST/ALT, TC and TG values are given in Table 1. The overweight patients showed significant increase in all parameters compared to healthy subjects. Furthermore, data analysis of obese patients suggests that they have higher score of all parameters compared to healthy subjects or overweight patients. The USG image of liver of one each of a healthy subject, overweight patient and obese patient are presented in Fig 8.The increased level of ALT, AST, TG and TC, AST/ALT ratio and ultrasound images of overweight and obese patientsclearly demonstrates fatty infiltration in livercells.

**Fig 9:**
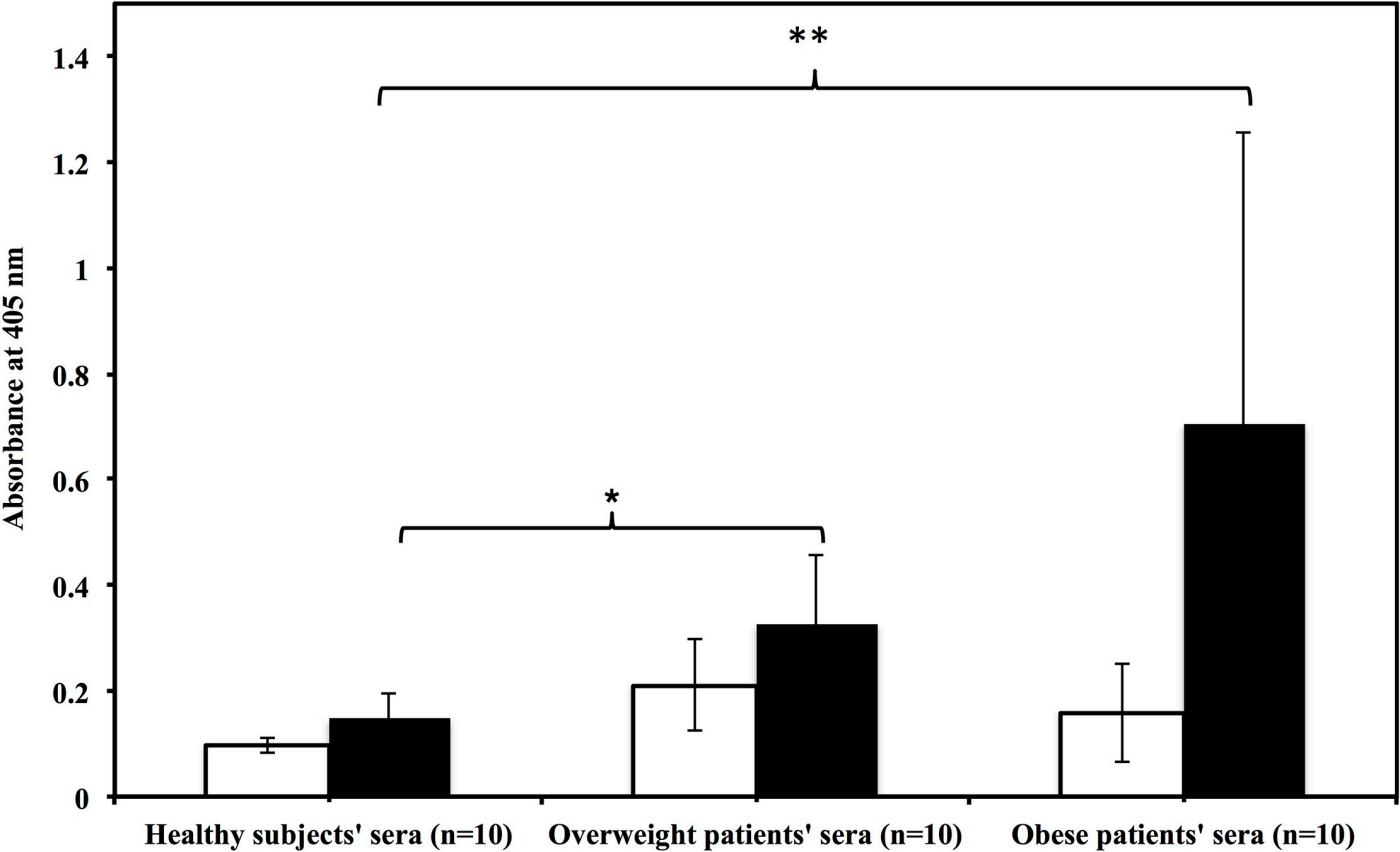
Direct binding ELISA of 1:100 diluted sera of overweight and obese patients with native HSA (□) and fructosylated-HSA-AGE (■). Sera obtained from healthy human subjects served as control. Microtitre plates were coated with respective antigens (10 μg/ml). The histogram represents mean ± SD value of 10 subjects from each group. *p< 0.05 with fructosylated-HSA-AGE in healthy vs overweight; **p < 0.05 with fructosylated-HSA-AGE in healthy vs obese.

Direct binding ELISA was carried out to determine the level of autoantibodies against fructosylated-HSA-AGE in sera of healthy subjects, overweight patients and obese patients and the results are shown in Fig 9. The data suggest that obese patients have highest level of circulating autoantibodies against fructosylated-HSA-AGE followed by overweight patients. Autoantibodies against fructosylated-HSA-AGE in healthy subjects sera were minimum.

### Specificity of serum autoantibodies in obese and overweight patients

The specificity of autoantibodies against fructosylated-HSA-AGE in some selected sera (which showed highest binding in direct binding ELISA) of obese and overweight patients was tested by inhibition ELISA. Data presented in Table 4 suggests that autoantibodies in sera of obese and overweight patients are quite specific which is in line with the results of direct binding ELISA. IgG isolated from the selected sera of obese and overweight patients maintained the specificity pattern (Table 5).

**Table 4:** Inhibition ELISA of serum autoantibodies in obese and overweight patients.

**Obese patients**		
**Serum no.**	**Maximum percent inhibition at 20 μg/ml**	
	**Native HSA**	**Fructosylated-HSA-AGE**
01	20.87%	47.1%
03	22.9%	44%
08	30.6%	43.7%
10	19.4%	42.7%
Mean ± SD	23.44±4.98%	44.375±1.899%
**Overweight patients**		
**Serum no.**	**Maximum percent inhibition at 20 μg/ml**	
	**Native HSA**	**Fructosylated-HSA-AGE**
05	20.7%	30.8%
07	18.4%	29.4%
Mean ± SD	19.55±1.626%	30.1±0.989%

Microtitre plates were coated with 10μg/ml of each native HSA and fructosylated-HSA-AGE

**Table 5:** Inhibition ELISA of IgG purified from selected sera of obese and overweight patients.

**Obese patients**			
**Serum no.**	**Maximum percent inhibition at 20** μ**g/ml**		
	**Native HSA**		**Fructosylated-HSA-AGE**
01	19.5%		53.8%
03	32.9%		59.9%
08	33.5%		56.3%
10	26.5%		61.3%
Mean ± SD	28.1±6.55%		57.825±3.411%
**Overweight patients**			
**Serum no.**	**Maximum percent inhibition at 20 μg/ml**		
	**Native HSA**		**Fructosylated-HSA-AGE**
05	25.5%		30.8%
07	17.8%		29.4%
Mean ± SD	21.65±5.444%		38.9±3.67%

Microtitre plates were coated with 10μg/ml of each native HSA and fructosylated-HSA-AGE

## Discussion

The consolidated effects of increased food consumption, low energy consumption, high blood sugar, high cholesterol and increased reactive oxidative stress can increase the formation of reactive intermediates and AGEs in obesity. The current dietary movement of fructose is likely to affect in vivo fructose concentrations and their metabolites in tissues and blood [38]. A couple of examinations have recommended that fructose and metabolites of fructose go about as endogenously produced toxins [2]. The intramuscular fructosylated-HSA-AGEs, which contains exogeneousfructose is taken up by hepatocytes via glucose transporters, Glut2 and Glut8 [16,39]. This fructose is converted into fructose-1-phosphate (F1P) by fructokinase. The phosphotrioses created from F1P by the action of aldolase B can be changed over either to lactate, glucose, or fatty acids [16]. Intramuscular injection of fructose results in about a threefold increase in hepatic dihydroxyacetone phosphate and glyceraldehyde-3-phosphate concentration [40]. In physiological conditions, the lipogenic pathway is minor but it turns out to be exceptionally dynamic after an intense fructose load as the motion of fructose carbons into lipogenic precursors increases [41]. High utilization of fructose causes saturation of the glycolytic pathway and collection of glycolysis intermediates, which can be changed over to glycerol-3-phosphate utilized in triglyceride (TG) formation [16].Recent, studies recommend that high AGE levels fuel liver damage, aggravation and fibrosis by means of oxidative stress, and HSC activation [42,43]. Endogenously formed CML accumulation with steatosis has been demonstrated in fatty livers of obese individuals [28]. Non-enzymatically glycated proteins are immunologically active inducing immune response [44]. The existence of autoimmune features in NASH has been reported, however its criticalness is indistinct [45]. Our previous findings have shown that concentrations of fructose have great impact on structure of HSA [31].The present investigation shows that fructosylated-HSA-AGE is hepatotoxic and immunologically dynamic and can incite reaction, which may worsen the liver damage pursued by liver harm.

Fructosylated-HSA-AGE was found to be a potent immunogen when injected into rabbits and induce high-titer antibodies as compared to native HSA. It indicates that fructose induced modifications on HSA have contributed to its immunogenicity, probably due to generation of neo-epitopes. It may be recalled that lysine enhances the immunogenicity of HSA [46]. Many studies also suggests that macromolecules undergo structural alterations upon glycation/fructosylation, resulting in neo-epitopes formation on macromolecule,which are recognized by the immune system as a foreign body and thus causing antibody responses [44,46,47]. The antigenic specificity on affinity purified anti-native HSA and anti-fructosylated-HSA-AGE IgGs pointed that immune response was established faster and more specifically in the animals that were immunized with fructosylated-HSA-AGE as compared to native HSA.

Fructose modification of HSA resulted in fructosylated-HSA-AGEs. These AGEs were deleterious and toxic. A few investigations have made it sufficiently evident that AGEs are engaged in the generation of oxidative stress, which intensify liver damage through liver inflammation, HSCs activation and amalgamation of extracellular matrix (ECM), causing liver fibrosis [48,49,50]. Histopathology results showed that rabbits injected with fructosylated-HSA-AGE developed signs of mild fibrosis with steatosis, portal inflammation, lymphatic infilteration and binucleation of hepatocytes. These outcomes were steady with the entrenched idea showing the pro-fibrotic [51,52], pro-inflammatory [53], and pro-oxidant [54,55] impacts of AGEs on liver and different tissues exhibiting its malicious role. One vital mechanism through which AGEs may add to tissue damage and fibrosis is the actuation as well as enlistment of myofibroblasts. In liver, AGEs increase recruit more numbers ofmyofibroblasts; and this might beattributed to worsening of liver fibrosis seen in animals injected with AGEs [56]. AGEs have additionally been appeared to add to the pathogenesis of tissue fibrosis through the generation of oxidative stress by means of enactment of nicotinamide adenine dinucleotide phosphate oxidase [57]. In vitro examinations have likewise demonstrated the capacity of AGEs to fundamentally enhance HSC responsive ROS generation [58]. AGEs and ROS can take an interest in an endless loop in which ROS advance the arrangement of AGEs, which drives further ROS generation, prompting expanded AGE collection and tissue injury.Moreover, fructosylated-HSA-AGE group animals showed increase in parameters of liver function (ALT, AST, bilirubin) and dyslipedimia (TC and TG) than those of the native HSA group animals. Taken together, our data indicated that fructosylated-HSA-AGE group rabbits showed some characteristics of NAFLD/NASH.During hepatic fibrosis, collagen protein types I and III proliferate and collagen formation increases [37]. It has been demonstrated that AGE-bovine serum albumin essentially expanded hepatic fibrosis as proved by expanded collagen content [59]. Our outcomes are additionally in congruity with the past examinations demonstrating the deposition of collagen fibres in the rabbits immunized with fructosylated-HSA-AGE.

A report suggests that with AST/ALT < 1, the liver diseases are often correlated to obesity and high cholesterol [25]. Ultrasonography, which should form part of the assessment of chronically raised transaminases, showing fatty infiltration.A comparative study on ALT, AST, AST/ALT ratio, bilirubin, TC and TG levels of healthy subjects and obese and overweight patients suggest that obese patients tend to have higher scores of above parameters compared to healthy subjects or overweight patients. Furthermore, the possible involvement of fructosylated-HSA-AGE in fatty liver disease was probed. The role of fructosylated-HSA-AGE as a potential immunogen was studied by analyzing sera of obese and overweight patients and healthy subjects’ for autoantibodies against fructosylated-HSA-AGE. The binding of circulating serum autoantibodies of obese patients, overweight patients and healthy subjects’ with native HSA and fructosylated-HSA-AGE was studied by direct binding ELISA. Forty percent sera of obese patients’ showed preferentially high binding with fructosylated-HSA-AGE. Only twenty percent of overweight patients’ sera showed preference for fructosylated-HSA-AGE. Healthy human sera were nearly devoid of autoantibodies that could bindfructosylated-HSA-AGE.

The strong binding of autoantibodies from obese and overweight patients to fructosylated-HSA-AGE points towards the involvement of modified lysine residues of albumin in the fatty liver disease. Autoantibodies detected in obese and overweight patients may be due to the generation of neo-epitopes on the HSA because of fructosylation.Fructose ingestion increases genes encoding lipogenic enzymes via activation of SCAP/SREBP pathway in the liver in a controlled manner. Excessive amount of fructose consumption leads to generation of AGEs, which results in a change, evoking delayed initiation of SCAP protein by restraining its degradation [60]. Subsequently there is overactivation of lipogenesis, which may change the lipid profiles. Thus, fructose is an important risk factor in the initiation and development of NAFLD and progression to NASH.

## Conclusion

From our data it may be concluded that fructose overconsumption may contribute in the development of obesity and dyslipidemia. Furthermore, it can rapidly form advanced glycation end products, which are hepatopathic as well as immunogenic.

## Abbreviations

ECM: extracellular matrix
NAFLD: non alcoholic fatty liver disease
NASH: non alcoholic steatohepatitis
HAS: human serum albumin
AGE: advanced glycation end products
ROS: reactive oxygen species
USG: ultrasonography
IgG: immunoglobulin G
ALT: alanine aminotransferase
AST: aspartate aminotransferase
TG: triglyceride
TC: total cholesterol
SREBP: sterol regulatory element-binding protein
SCAP: SREBP cleavage-activating protein
